# Association between obesity and education level among the elderly in Taipei, Taiwan between 2013 and 2015: a cross-sectional study

**DOI:** 10.1038/s41598-020-77306-5

**Published:** 2020-11-20

**Authors:** Tsai-Hao Hsieh, Jason Jiunshiou Lee, Ernest Wen-Ruey Yu, Hsiao-Yun Hu, Shu-Yi Lin, Chin-Yu Ho

**Affiliations:** 1 Department of Family Medicine, Taipei City Hospital, Yangming Branch, Taipei, Taiwan; 2 Institute of Public Health, National Yang-Ming University, Taipei, Taiwan; 3 University of Taipei, Taipei, Taiwan; 4 Faculty of Medicine, National Yang-Ming University, Taipei, Taiwan; 5 Department of Education and Research, Taipei City Hospital, Taipei, Taiwan; 6 Department of Psychology, Soochow University, Taipei, Taiwan

**Keywords:** Diseases, Risk factors

## Abstract

The inverse association between obesity and education level has been demonstrated in many developed countries; however, few studies have investigated obesity in geriatric populations. This cross-sectional analysis explored the association between geriatric obesity and education level, along with other demographic characteristics in Taipei, Taiwan between 2013 and 2015. Taipei citizens ≥ 65 years (aborigines ≥ 55 years) were recruited to participate in the elderly health examination programme. Logistic regression was applied to analyse the relationship between obesity (defined as body mass index ≥ 27 kg/m^2^ in Taiwan) and education level among men and women after controlling for age, race, income status, and smoking status. A total of 28,092 men and 31,835 women were included in the final analysis. Compared to those with education years ≥ 16, older men and women with education years ≤ 12 had higher odds of being obese. The odds ratios increase as years of education decrease, and the trend is more pronounced among women. Aborigines had much higher chances of being obese among men and women, while there were no differences by income status. The results clarified the factors related to obesity in the elderly, and will be useful for authorities working to improve health outcomes among this population.

## Introduction

The United Nations (UN) and the World Health Organization (WHO) defined ‘ageing’, ‘aged’, and ‘super-aged’ societies as populations where the proportion of people aged 65 years and older ranges from 7 to 14%, > 14%, and > 20%, respectively. Many developed countries are gradually moving towards aged and super-aged societies, including Taiwan. Taiwan advanced from an ageing society to an aged society in only 25 years (1993–2018), much faster than other countries^1–3^. In Taipei, especially, the population aged 65 and over grew much faster than the population in the rest of Taiwan; therefore, as early as 2014, Taipei had become an aged society^4^. It is estimated that Taiwan will enter the era of super-aged society in 2026, and the Taiwan government needs to adapt quickly to this new reality^1^.

At the same time, the worldwide prevalence of obesity in adults has nearly tripled since 1975; 39% of adults ≥ 18 years old were overweight, and 13% were obese in 2016^5^. The Nutrition and Health Survey in Taiwan (NAHSIT) reported that the prevalence of obesity among Taiwanese adults was 22.8% in 2013–2016, higher than that in 1993–1996 (11.5%) and in 2005–2008 (17.9%). Geriatric obesity has also increased over time. The prevalence of obesity among older adults in Taiwan was 18.9% in 1993–1996, 22.2% in 2005–2008, and 22.8% in 2013–2016^6^. All these statistics testify to the gravity of geriatric obesity as a forthcoming major health issue in Taiwan.

The current evidence shows that, while overweight may serve as a protection against the risk of death in the general community-dwelling elderly population, obesity appears to be associated with a statistically significant increase in risk of death among the elderly^7^. Obesity also has positive correlations with a plethora of chronic diseases and medical problems, including hypertension, dyslipidemia, type 2 diabetes, coronary heart disease, and even the incidence of cancers^8–11^, resulting in significant health burdens.

Previous reports have shown differences in the prevalence of obesity among adults in the United States by sex, age group, race, household income, education level, smoking status, and urbanisation level^12,13^. Adults living in nonmetropolitan statistical areas had a significantly higher prevalence of obesity and severe obesity compared with those living in large metropolitan statistical areas^12^. Both women and men who were college graduates had lower prevalence of obesity than those with less education^12,13^. In a report from the Organization for Economic Co-operation and Development (OECD), developed countries, such as Australia, Canada, and England, showed similar results to the US in that the prevalence of obesity was lower among those with higher education levels^14^. Data from Korea National Health and Nutrition Examination Survey (KNHANES) in 1998–2005 and 2010–2012 disclosed that education was positively associated with obesity in men, i.e., people with higher education levels were more likely to be obese, but the results might differ in models with and without interaction-effect terms of independent variables. The patterns differed substantially between sexes in Korea, and women with higher education levels had a significantly lower possibility of being obese, the same as other developed countries^14–16^. A report on health inequalities in Taiwan also found that differences in the rates of overweight and obesity among women were substantial. Women with less than junior school education had a far higher rate of overweight or obesity than other groups, and groups with higher education levels had a lower prevalence of overweight or obesity^17^. Another Taiwanese study showed morbid obesity to be associated with low socioeconomic status (SES)^18^.

Previous studies placed less emphasis on obesity among elderly populations. One study comparing the elderly in Japan and the US showed that each year of education reduced the relative likelihood of obesity by 5–9%. This study had some limitations, however, including insufficient numbers of obesity among Japanese older adults and the self-reported data used in the analysis^19^. Since geriatric obesity will cause an increase in government expenditure on healthcare in the near future, there is an urgent need for identifying the associated risk factors. Evidence showed multiple pathways from SES (including education level, income, and occupation) to health, including differential exposure to chronic stress and its biological toll^20^. Establishing the correlation between geriatric obesity and education level will allow relevant authorities to develop effective interventions to reduce the prevalence of obesity. In this study, we performed a cross-sectional analysis to explore the association between geriatric obesity and education level, along with other demographic characteristics in Taipei, Taiwan.

## Materials and methods

This was a cross-sectional study using multivariate logistic regression analysis to evaluate the relationship between education level and obesity among the elderly. Data were collected from the elderly health examination programme in Taipei from 2013 to 2015. The elderly health examination programme is conducted annually in Taipei city and funded by the municipal government. The raw data were made available after applying to the Department of Health, Taipei City Government. This study was conducted by analysing datasets, and the raw data were de-identified, therefore the Research Ethics Committee agreed to waive the informed consent due to minimal risk within the study. The study was approved by the Taipei City Hospital Research Ethics Committee with the case number TCHIRB-10805022-W. All methods were carried out in accordance with relevant guidelines and regulations of the Taipei City Hospital Research Ethics Committee.

Taipei citizens older than 65 years old are qualified to sign up for the annual elderly health examination at no cost. As for the citizens with aboriginal identity, criteria of registration were expanded to 55 years or older. All the health examinations were conducted at the contracted hospitals in Taipei. The participants underwent some of the following check-up items: physical examination, blood test, urine analysis, chest X-ray, electrocardiogram, and abdominal ultrasonography. Well-trained interviewers also performed the AD8 dementia screening and depression screening test. Participants’ background information, such as education level, smoking status, and past medical history, was obtained using a questionnaire designed by the Department of Health, Taipei City Government. Height and weight were measured similarly in all the contracted hospitals using standardised techniques, and participants were asked to remove their shoes before the measurements were taken. The devices used in the measurement of body height and weight were all calibrated regularly under the hospitals’ standard operating procedures. Obesity in this study was defined as body mass index (BMI) ≥ 27 kg/m^2^, according to the Ministry of Health and Welfare of Taiwan. The definitions of obesity (BMI ≥ 30 kg/m^2^) and overweight (BMI: 25 to < 30 kg/m^2^) by WHO are based primarily on criteria derived from studies involving populations of European origin. It has been suggested that the BMI cut-off point (≥ 30 kg/m^2^) might be too high for Asians, thereby underestimating associated health risks^21,22^. Therefore, the Ministry of Health and Welfare of Taiwan defines obesity (BMI ≥ 27 kg/m^2^) and overweight (BMI: 24 to < 27 kg/m^2^) using local statistic results. Normal BMI is defined as 18.5 to < 24 kg/m^2^ in Taiwan, and BMI < 18.5 kg/m^2^ is considered underweight^6^.

We included all participants from 2013 to 2015 in this study. For participants having health examinations in more than one year, only the data from the latest year were included in further analysis. Demographic variables included sex, age, race, income status, smoking status, and education level because previous studies have shown differences in the prevalence of obesity based on these factors^12–16,19^. We excluded individuals with missing or wrong data, or showing other extreme values, for example: age > 110 years, body weight > 120 kg or < 20 kg, body height > 200 cm or < 120 cm, and BMI > 50 kg/m^2^ or < 10 kg/m^2^.

The original questionnaire categorised education level as unknown, illiterate, self-study, primary school, junior high school, junior vocational school, senior high school, senior vocational school, 5-year junior college, 2-year or 3-year junior college, university, and graduate institute. In addition, ‘complete’ and ‘incomplete’ were used to describe the level of education in more detail. To simplify and make all the education levels comparable, they were transformed to years of education in further analysis, and collapsed into the following five categories: no more than 6 years (below or equivalent to primary school graduate), 7 to 9 years (equivalent to complete or incomplete junior high school), 10 to 12 years (equivalent to complete or incomplete senior high school), 13 to 15 years (equivalent to junior college graduate or some college), and no less than 16 years (equivalent to university graduate or graduate institute). The five categories were based on the Taiwanese education process, which mainly includes 6 years at a primary school, 3 years at a junior high school, 3 years at a senior high (or vocational) school, and 4 years at a university (or college), and most people follow this process.

Participants’ race was categorised as aborigine or non-aborigine according to the household registration records of the Department of Household Registration, Ministry of the Interior. Income status was divided into normal, middle-income, middle/low-income, and low-income households from the raw data according to the records from the Department of Social Welfare, Taipei City Government. The Ministry of Health and Welfare in Taiwan defines normal and lower-income households based on monthly living expenses by different areas in Taiwan^23^. In this study, participants from middle-income, middle/low-income, and low-income households were collapsed into one group—lower-income households—to clarify whether lower income is related to obesity. There were four options for smoking status in the original questionnaire: smoking every day, smoking only after meals, smoking only while socialising or at the invitation of friends, and never smoke. While the former two were categorised as signifying a current smoker in the current study, smoking only while socialising or at the invitation of friends was categorised as signifying an occasional smoker, and the final option was categorised as signifying a non-smoker. These variables were all contained in the raw data obtained from the Department of Health, Taipei City Government.

Multivariate logistic regression was applied to analyse the association between education level and obesity in both sexes after controlling for all other confounding factors, including age, race, income status, and smoking status. The results mainly contained prevalence of obesity by demographic characteristics and the odds ratio (OR) estimates of being obese with 95% Wald confidence interval (95% CI). The reference groups of each variable were participants with no less than 16 years of education, non-aborigine, a normal income status, and non-smokers. To ensure the homogeneity of results between different years of data collection, participants were divided into three groups according to the year in which their data had been obtained, and multivariate logistic regression regarding education level was conducted for these three subgroups. Another subgroup analysis was conducted by participants’ age, and all participants were stratified into three cohort groups: ≤ 70, 71–80, and > 80 years old. All analyses were conducted using SAS version 9.4 (SAS Institute Inc).

## Results

**Figure 1:**
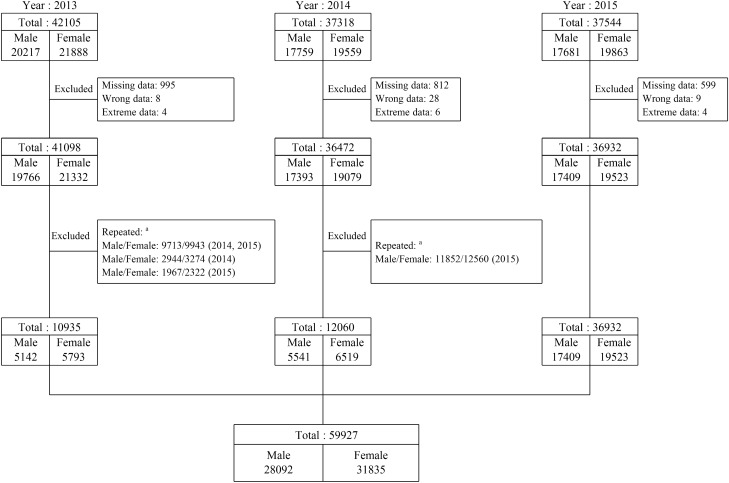
Data acquisition flow diagram. ^a^For participants having health examinations in more than one year, only the data of the latest year was included in further analysis.

The initial database included 42,105, 37,318, and 37,544 original data from 2013, 2014, and 2015, respectively. After excluding data with missing information and erroneous values, data for the same participants from different years, and participants meeting the exclusion criteria, a total of 28,092 male participants with a mean age of 76.8 years and 31,835 female participants with a mean age of 74.2 years were included in the final analysis. The flow diagram in Fig. 1 depicts data process flow.

### Distribution of BMI in the sample

The sample sizes for enrolled older men and women by BMI, education level, income status, race, and smoking status appear in Table 1. All characteristics differed significantly by sex (*p* value < 0.001). Among older men in Taipei, the prevalence of obesity (BMI ≥ 27 kg/m^2^) and overweight (BMI: 24 to < 27 kg/m^2^) were 16.4% and 33.2%, respectively; most had a normal BMI (46.6%), and only a small proportion was underweight (3.8%). As for older women, the prevalence of obesity and overweight were 18.3% and 27.5%, respectively; similar to men, most women had a normal BMI (49.2%), and only 5.0% were underweight.

**Table 1:** Demographic characteristics of participants enrolled from the elderly health examination programme in Taipei, 2013–2015.

Characteristic	No. of participants (%)		*p* value^a^
	Men	Women	
All	28,092 (100)	31,835 (100)	
Age, years^b^	76.8 ± 7.80^c^	74.2 ± 6.89^c^	< 0.001
Body mass index (kg/m^2^)^d^			
Underweight (< 18.5)	1059 (3.8)	1583 (5.0)	< 0.001
Normal (18.5– < 24)	13,088 (46.6)	15,656 (49.2)	
Overweight (24– < 27)	9326 (33.2)	8753 (27.5)	
Obesity (≥ 27)	4619 (16.4)	5843 (18.3)	
Education level, years			
≤ 6	5425 (19.3)	11,531 (36.2)	< 0.001
7–9	2818 (10.0)	5146 (16.2)	
10–12	6315 (22.5)	7582 (23.8)	
13–15	3320 (11.8)	2851 (9.0)	
≥ 16	10,214 (36.4)	4725 (14.8)	
From lower-income household^e^			
No	27,216 (96.9)	31,238 (98.1)	< 0.001
Yes	876 (3.1)	597 (1.9)	
Race			
Non-aborigine	27,790 (98.9)	31,255 (98.2)	< 0.001
Aborigine	302 (1.1)	580 (1.8)	
Smoking^f^			
Non-smoker	25,408 (90.4)	31,497 (98.9)	< 0.001
Occasional smoker	754 (2.7)	125 (0.4)	
Current smoker	1930 (6.9)	213 (0.7)	

^a^*p* values were obtained from t-test for numerical variables and from Chi-squared test for categorical variables.

^b^The elderly health examination programme in Taipei enrolled citizens older than 65 (aborigines older than 55), and data with age < 65 (aborigines’ age < 55) or > 110 was excluded.

^c^Values are presented as mean ± standard deviation.

^d^Body mass index is categorised according to the Ministry of Health and Welfare of Taiwan.

^e^Lower-income households include middle-income, middle/low-income, and low-income households.

^f^Non-smoker includes participants who have never smoked, occasional smoker includes participants smoking only while socialising or at the invitation of friends, and current smoker includes participants smoking every day and smoking only after meals.

### Distribution of education level, aborigines, income status, and smoking status in the sample

The education level appeared unequal between men and women in this surveyed cohort. There were mostly older men in Taipei with no less than 16 years of education (36.4%). In addition, 19.3% of the men fell in the education level category of no more than six years, 10.0% in the category of 7–9 years, 22.5% in the category of 10–12 years, and 11.8% in the category of 13–15 years. In contrast, there were mostly older women with no more than six years of education (36.2%), with 16.2% having 7–9 years, 23.8% having 10–12 years, 9.0% having 13–15 years, and only 14.8% having no less than 16 years of education. The smoking status also showed a difference between both sexes, with current smokers accounting for 6.9% of older men but only 0.7% of older women. The proportion of aborigines was 1.1% among men and 1.8% among women in this study. Both men and women with lower income status occupied a small proportion of all participants; 3.1% of men and 1.9% of women were from lower-income households.

### Prevalence of obesity and odds ratio estimate by demographic characteristics

The prevalence of obesity and odds ratio estimates by demographic characteristics appear in Table 2. In general, the prevalence of obesity increased as years of education decreased among the elderly, and the same trend applied for both men and women. The prevalence of obesity was 19.2%, 18.5%, 17.1%, 14.2%, and 14.7% for men with no more than six years, 7–9 years, 10–12 years, 13–15 years, and no less than 16 years of education, respectively. Compared with those having years of education ≥ 16, older men in Taipei having fewer years of education had higher odds of being obese, and the odds ratio (OR) was 1.436 for those having years of education ≤ 6 (95% CI 1.314–1.570, *p* < 0.001), 1.363 for 7–9 years of education (95% CI 1.219–1.523, *p* < 0.001), and 1.213 for 10–12 years of education (95% CI 1.113–1.322, *p* < 0.001). The odds ratios of the above three categories had statistical significance. The OR for 13–16 years of education was non-significant at 0.972 (95% CI 0.868–1.087, *p* = 0.614). Similar results were found in older women. The prevalence of obesity was 24.1%, 19.0%, 15.1%, 13.0%, and 12.1% for women with no more than six years, 7–9 years, 10–12 years, 13–15 years, and no less than 16 years of education, respectively. The OR was 2.278 for years of education ≤ 6 (95% CI 2.062–2.517, *p* < 0.001), 1.680 for 7–9 years of education (95% CI 1.500–1.881, *p* < 0.001), 1.278 for 10–12 years of education (95% CI 1.146–1.424, *p* < 0.001), and 1.087 for 13–16 years of education (95% CI 0.945–1.251, *p* = 0.241) compared with those having ≥ 16 years of education. Although there was no statistically significant difference between individuals with 13–16 and ≥ 16 years of education, it was evident that education level had a negative correlation with obesity among the elderly in Taipei, especially among women.

**Table 2:** Prevalence of obesity and odds ratio (OR) by age, education level, income status, race, and smoking status among men and women enrolled from the elderly health examination programme in Taipei, 2013–2015.

Characteristic	Men			Women		
	Prevalence, %	OR (95% CI)^a^	*p* value	Prevalence, %	OR (95% CI)^a^	*p* value
Age^b^		0.977 (0.972–0.981)^c^	< 0.001		0.999 (0.995–1.003)	0.644
Education level, years						
≤ 6	19.2	1.436 (1.314–1.570)^c^	< 0.001	24.1	2.278 (2.062–2.517)^c^	< 0.001
7–9	18.5	1.363 (1.219–1.523)^c^	< 0.001	19.0	1.680 (1.500–1.881)^c^	< 0.001
10–12	17.1	1.213 (1.113–1.322)^c^	< 0.001	15.1	1.278 (1.146–1.424)^c^	< 0.001
13–15	14.2	0.972 (0.868–1.087)	0.614	13.0	1.087 (0.945–1.251)	0.241
≥ 16	14.7	1 [Reference]		12.1	1 [Reference]	
From lower-income household						
No	16.5	1 [Reference]		18.3	1 [Reference]	
Yes	14.5	0.907 (0.746–1.102)	0.325	21.3	1.061 (0.868–1.297)	0.566
Race						
Non-aborigine	16.1	1 [Reference]		18.0	1 [Reference]	
Aborigine	43.7	2.936 (2.316–3.722)^c^	< 0.001	38.3	2.616 (2.184–3.133)^c^	< 0.001
Smoking						
Non-smoker	16.3	1 [Reference]		18.3	1 [Reference]	
Occasional smoker	19.4	1.079 (0.895–1.300)	0.426	20.0	0.830 (0.529–1.304)	0.419
Current smoker	16.6	0.861 (0.758–0.978)^c^	0.022	20.7	0.874 (0.622–1.229)	0.439

*OR* odds ratio, *CI* confidence interval.

^a^OR and 95% CI were estimated with a multivariate logistic regression model that included all 5 characteristics presented in this table.

^b^The OR of age described odds ratio of being obese with each additional year.

^c^Statistically significant results.

The prevalence of obesity was 16.6% for current male smokers, 19.4% for male occasional smokers, and 16.3% for male non-smokers, but the OR was 0.861 (95% CI 0.758–0.978, *p* = 0.022) among current male smokers and 1.079 (95% CI 0.895–1.300, *p* = 0.426) among male occasional smokers compared with male non-smokers. The prevalence is the outcome of descriptive statistics, and the odds ratio is the outcome of inferential statistics after controlling for other variables, hence there is no absolute correlation between the two, although the latter is more rigorous. The prevalence of obesity was 20.7% among current female smokers, 20.0% among female occasional smokers, and 18.3% among female non-smokers, but the odds of being obese showed no statistically significant difference between the former two and the latter one subgroups. Among aborigines, both older men and women had much higher odds of being obese; while 43.7% of aborigine men (OR 2.936, 95% CI 2.316–3.722, *p* < 0.001) and 38.3% of aborigine women (OR 2.616, 95% CI 2.184–3.133, *p* < 0.001) were obese, only 16.1% and 18.0% of their counterparts had obesity. For each additional year of older men, the odds of being obese reduced a bit but were still statistically significant (OR 0.977, 95% CI 0.972–0.981, *p* < 0.001). On the other hand, older women’s age was not related to the chance of being obese in this study. Individuals from lower-income households, regardless of sex, did not differ in terms of their odds of being obese, compared to those from normal households.

### Subgroup analyses by year of data and participants’ age

Subgroup analyses by the year of data and by participants’ age appear in Tables 3 and 4. Table 3 shows a larger number of participants in 2015, compared to 2014 and 2013. This is because, in case of repeated participation, only data from the most recent year would be used. Figure 1 also shows that the repeated participants account for a large proportion of all participants (30,163/41,098 in 2013 and 24,412/36,472 in 2014). Among both sexes, individuals with higher education levels held a slightly higher proportion in 2015 than in 2014, with a slightly higher proportion also observed in 2014 compared to 2013. As shown in Table 4, the younger groups had higher average education levels, and higher prevalence of obesity among participants with all education levels. All analyses showed the same trend: that participants with higher education levels had lower prevalence of obesity and lower odds of being obese.

**Table 3:** Prevalence of obesity and odds ratio (OR) by education level in each year among men and women enrolled from the elderly health examination programme in Taipei, 2013–2015.

Year	2013						2014						2015					
Numbers	Men (N = 5142)			Women (N = 5793)			Men (N = 5541)			Women (N = 6519)			Men (N = 17,409)			Women (N = 19,523)		
Education level, years	No. (%)^a^	Prevalence of obesity, %	OR (95% CI)^b^	No. (%)^a^	Prevalence of obesity, %	OR (95% CI)^b^	No. (%)^a^	Prevalence of obesity, %	OR (95% CI)^b^	No. (%)^a^	Prevalence of obesity, %	OR (95% CI)^b^	No. (%)^a^	Prevalence of obesity, %	OR (95% CI)^b^	No. (%)^a^	Prevalence of obesity, %	OR (95% CI)^b^
≤ 6	1287 (25.0)	20.4	1.379 (1.134–1.677)^c^	2650 (45.7)	26.8	2.257 (1.764–2.888)^c^	1239 (22.4)	18.5	1.404 (1.152–1.710)^c^	2586 (39.7)	23.4	2.062 (1.633–2.604)^c^	2899 (16.7)	19.0	1.449 (1.289–1.629)^c^	6295 (32.2)	23.2	2.254 (1.989–2.553)^c^
7–9	606 (11.8)	20.0	1.340 (1.050–1.710)^c^	925 (16.0)	21.5	1.679 (1.271–2.217)^c^	585 (10.6)	21.5	1.723 (1.357–2.187)^c^	1088 (16.7)	19.1	1.602 (1.234–2.080)^c^	1627 (9.3)	16.8	1.232 (1.062–1.428)^c^	3133 (16.1)	18.2	1.656 (1.437–1.909)^c^
10–12	1146 (22.3)	18.7	1.213 (0.991–1.486)	1239 (21.4)	17.9	1.314 (1.003–1.723)^c^	1254 (22.6)	16.3	1.170 (0.958–1.428)	1502 (23.0)	15.1	1.207 (0.936–1.557)	3915 (22.5)	16.9	1.217 (1.092–1.357)^c^	4841 (24.8)	14.4	1.263 (1.104–1.444)^c^
13–15	547 (10.6)	13.9	0.863 (0.653–1.141)	376 (6.5)	13.0	0.893 (0.612–1.302)	576 (10.4)	13.9	0.965 (0.736–1.266)	547 (8.4)	13.7	1.093 (0.792–1.508)	2197 (12.6)	14.3	1.001 (0.872–1.149)	1928 (9.9)	12.9	1.126 (0.950–1.336)
≥ 16	1556 (30.3)	16.2	1 [Reference]	603 (10.4)	14.4	1 [Reference]	1887 (34.0)	14.6	1 [Reference]	796 (12.2)	12.6	1 [Reference]	6771 (38.9)	14.4	1 [Reference]	3326 (17.0)	11.5	1 [Reference]

*OR* odds ratio, *CI* confidence interval.

^a^Indicates the number and percentage of participants with different education levels.

^b^OR and 95% CI were estimated with a multivariate logistic regression model that were controlled for age, income status, race, and smoking status. ^c^Statistically significant results, *p* value < 0.05.

**Table 4:** Prevalence of obesity and odds ratio (OR) by education level in age grouping ≤ 70, 71–80, and > 80 years old among men and women enrolled from the elderly health examination programme in Taipei, 2013–2015.

Age, years	≤ 70						71–80						> 80					
Numbers	Men (N = 5914)			Women (N = 9188)			Men (N = 12719)			Women (N = 16,770)			Men (N = 9459)			Women (N = 5877)		
Education level, years	No. (%)^a^	Prevalence of obesity, %	OR (95% CI)^b^	No. (%)^a^	Prevalence of obesity, %	OR (95% CI)^b^	No. (%)^a^	Prevalence of obesity, %	OR (95% CI)^b^	No. (%)^a^	Prevalence of obesity, %	OR (95% CI)^b^	No. (%)^a^	Prevalence of obesity, %	OR (95% CI)^b^	No. (%)^a^	Prevalence of obesity, %	OR (95% CI)^b^
≤ 6	765 (12.9)	30.2	1.397 (1.143–1.706)^c^	2125 (23.1)	30.8	2.390 (2.030–2.814)^c^	2719 (21.4)	18.6	1.466 (1.294–1.661)^c^	6709 (40.0)	22.9	2.231 (1.934–2.574)^c^	1941 (20.5)	15.7	1.330 (1.127–1.570)^c^	2697 (45.9)	21.7	2.116 (1.562–2.866)^c^
7–9	463 (7.8)	25.1	1.195 (0.931–1.534)	1183 (12.9)	22.7	1.528 (1.253–1.863)^c^	1267 (10.0)	19.2	1.497 (1.277–1.755)^c^	2908 (17.3)	18.1	1.717 (1.463–2.016)^c^	1088 (11.5)	14.9	1.233 (1.010–1.506)^c^	1055 (18.0)	17.3	1.604 (1.153–2.231)^c^
10–12	1272 (21.5)	25.8	1.321 (1.114–1.565)^c^	2534 (27.6)	17.6	1.268 (1.071–1.503)^c^	2818 (22.1)	16.0	1.216 (1.071–1.380)^c^	3735 (22.3)	12.9	1.190 (1.014–1.396)^c^	2225 (23.5)	13.4	1.089 (0.924–1.282)	1313 (22.3)	16.4	1.506 (1.089–2.082)^c^
13–15	802 (13.6)	20.6	1.131 (0.921–1.387)	1086 (11.8)	15.5	1.138 (0.915–1.411)	1294 (10.2)	12.4	0.870 (0.727–1.042)	1406 (8.4)	10.9	0.991 (0.807–1.217)	1224 (13.0)	11.9	0.947 (0.772–1.162)	359 (6.1)	14.2	1.263 (0.834–1.911)
≥ 16	2612 (44.2)	19.8	1 [Reference]	2260 (24.6)	12.9	1 [Reference]	4621 (36.3)	13.3	1 [Reference]	2012 (12.0)	11.2	1 [Reference]	2981 (31.5)	12.5	1 [Reference]	453 (7.7)	11.5	1 [Reference]

*OR* odds ratio, *CI* confidence interval.

^a^Indicates the number and percentage of participants with different education levels.

^b^OR and 95% CI were estimated with a multivariate logistic regression model that were controlled for income status, race, and smoking status.

^c^Statistically significant results, *p* value < 0.05.

## Discussion

This study reveals the inverse association between obesity and education level among the elderly, which means that obesity prevalence and the odds of being obese increase with decreasing years of education. Compared with those having more than 16 years of education, the prevalence and the odds ratio showed a gradual but noticeable increment as years of education decreased. The trend was more apparent among women, and the gaps of prevalence and odds ratio between each subgroup of women were more significant than that for subgroups of men. According to the data released from the Department of Budget, Accounting and Statistics, Taipei City Government, the total population of Taipei over 65 years old was 399,182 (men: 181,035, women: 218,147) in 2015, and the proportion of men and women with an education level below or equivalent to primary school was 23.8% and 44.8%, equivalent to junior high school was 11.3%, and 14.5%, equivalent to senior high school was 21.2 and 19.8%, and equivalent to college or above was 43.8% and 20.9%, respectively^4^. Therefore, the educational inequality between older men and women did exist in Taipei, as it does in Taiwan^24^, which might be attributed to the socio-cultural context and the traditional attitudes towards gender roles at the time when they were studying. After inspecting the data of this study and the data from Taipei City Government in detail, it can be said that Taipei citizens with a higher education level might be more likely to participate in elderly health examinations. The actual statistics show that 43.8% of older men and 20.9% of older women in Taipei have an education level equivalent to or above college, and in this study the proportion of male and female participants with education years ≥ 13 was 48.2% and 23.8%, respectively. The lower education group presented the opposite trend, as there are 23.8% of older men and 44.8% of older women in Taipei having an education level below or equivalent to primary school, but the proportion of male and female participants with education years ≤ 6 in this study was only 19.3% and 36.2%, respectively. In Table 3, the proportion of participants with education years ≥ 16 in 2015 was greater than in 2013 and 2014, and it is known that repeated participants comprised more than half the participants in 2015, therefore supporting the aforementioned inference. This might indicate a higher level of health awareness among the more educated.

According to past index studies and recent literature that does not consider individuals’ ages, the results of developing countries and developed countries with regard to obesity prevalence have been distinct. A positive association between obesity and education level has been more common among men in lower-income countries, but an inverse association has been more common among women in lower-income countries and in both sexes in higher-income countries^12–16,25–28^. Taiwan is classified as a high-income economy by the World Bank and as an advanced economy by the International Monetary Fund^29,30^. The government of Taiwan calculated its own Human Development Index (HDI) using the criteria of the United Nations Development Programme (UNDP), and this HDI, having a value higher than 0.800—the cut-off for the categorisation into a very high HDI—at 0.911 in 2018, placed Taiwan in the category of very high human development countries^31,32^. The results of this study indicated that the associations mentioned above were not changed by the subjects’ age being limited to the elderly. Previous analyses of the United States have also shown that the prevalence of obesity among adults has been lower in the higher income group^13^, but similar results were not obtained in this study, and the difference might be attributed to the very low proportion of participants with a lower household income (3.1% in men and 1.9% in women).

The possibility of being obese decreased with age among older men but not older women in this study, but the study’s results did not display any causality between age and BMI in both sexes. The result of this cross-sectional analysis was influenced by the age distribution of all the participants and the possible cohort effect within different age groups. Much higher prevalence of obesity among the younger groups is noted in Table 4, and this phenomenon may be linked to the cohort effect in respect to improved nutritional intake, rather than age itself. Despite this, previous longitudinal cohort studies revealed that BMI, as well as body weight and height, all decline with age in the elderly. BMI declines especially after the age of 70 years, but the trend becomes statistically insignificant after the age of 90 years^33,34^.

Studies in the United States have shown that race has an apparent effect on obesity^3,12^. However, the literature on Taiwanese aborigines once showed that after controlling for other variables, regression analyses revealed few associations with increased risk of obesity in the aborigines^35^. This study also controlled for related variables, and still found that Taiwanese aborigines have a much higher possibility of being obese. Past literature on health disparities pointed out that even if education level and income were controlled, there were still other factors, such as the amount of wealth and debt, that could affect health outcomes among different race groups^20^. Therefore, to clarify the correlation between Taiwanese aborigines and obesity, more prospective and well-designed experiments are necessary.

In this study, older smoking men had less possibility of being obese, but older smoking women did not have the same tendency, which might be due to the low proportion of older smoking women. Occasional smokers, both men and women, showed no differences in terms of the possibility of being obese compared with non-smokers. A previous systematic review showed that even light and intermittent smoking carried higher risks of frailty and physical disability in older adults, and higher risks of cardiovascular disease, respiratory diseases, reproductive health concerns, lung cancer, and gastrointestinal cancers in the general population. The same review also showed that cardiovascular mortality and all-cause mortality were positively related to occasional smoking^36^. The correlation between smoking and body weight loss is well-established, but the physiological mechanisms are complex and incompletely understood. Body weight is determined by the balance of caloric intake and energy expenditure. Most literature agrees with the effects of cigarette smoking on body weight mediated by nicotine, and it is generally assumed that nicotine reduces body weight by increasing energy expenditure and reducing appetite^37,38^. It is worth noting that smoking increases insulin resistance and is associated with central fat accumulation, which increases the risk of metabolic syndrome and diabetes, resulting in an elevated risk of cardiovascular disease^38^. Previous literature has also pointed out that cigarette smoking has been the leading cause of preventable morbidity and mortality in the United States^39,40^. Therefore, the benefits of decreased body weight due to smoking do not overcome the associative risks.

This is one of the few studies focussing on the relationship between obesity and education level among the elderly, and the study also has several other strengths. Possible confounders mentioned by previous studies, including age, race, smoking status, and income status^12,13,25^, were all controlled in the current analysis. The results were stratified by sex because background information differed between men and women, and this would highlight any existing differences. Some earlier studies did find differences related to sex^26,27^, including studies conducted in South Korea^14–16^, a country with a similar cultural background to Taiwan. Subgroup analyses by data year and age grouping were also taken into account in this study.

There were several limitations to this study. Although this cross-sectional analysis revealed the inverse association between obesity and education level among the elderly, it was unable to elucidate the causal relationship between the two. The use of quasi-experimental designs or further long-term prospective cohort studies with individuals without obesity, including children, adolescents, adults, and the elderly, may assess the impact of education on BMI more precisely. Considering the data used in the current study, participants with extreme values for age (> 110 years), body weight (> 120 kg or < 20 kg), body height (> 200 cm or < 120 cm), and BMI (> 50 kg/m^2^ or < 10 kg/m^2^) were excluded from the final analysis, but it had little effect since the proportion of excluded participants was negligible. The original data for this study divided income status into only four categories: normal, middle-income, middle/low-income, and low-income households. Higher income has previously been associated with lower body weight among American women, with a U-shaped association for men in the US^13^. Thus, while we understand this to potentially have an impact on the results, there was insufficient information to further stratify participants from normal income households, which accounted for the majority of participants (96.9% of males and 98.1% of females). Apart from these, the results of this study might not be representative of general older adults in Taiwan or even in Taipei because selection bias was inevitable, and only Taipei citizens who got the information and signed up for the elderly health examination could be included in this study.

With the increasing prevalence of obesity in older adults, this study initially identified groups who were more susceptible to obesity. Improvement in diet, increases in physical activity, and a combination of dietary and physical activity interventions have proven effective in obesity prevention and weight management among the elderly, but access to healthy foods could be a barrier for older adults, especially those with low SES^41^. Physical and social environments vary by SES and affect the likelihood of individuals’ exposure to both health damaging conditions and health-protecting resources^20^. Education is associated with the acquisition of beliefs and knowledge, enabling people to integrate healthy behaviours into a coherent lifestyle, and giving them a sense of control over their health^42^. With this in mind, relevant government departments of public health could develop targeted empowerment programmes for such disadvantaged groups.

## Conclusions

This study primarily established the correlation between obesity and education level among the elderly. Like other developed countries, there was an inverse association between obesity and education level among the elderly in Taipei in 2013–2015; both older men and women with higher education levels have a lower possibility of being obese, and the trend is more pronounced among women. Meanwhile, Taiwanese aborigines have a much higher risk of obesity, and older smoking men have a relatively low risk. Further longitudinal studies with quasi-experimental designs may help to elucidate the causality between education level and obesity. Collecting measures of education level prior to being obese can decrease potential reverse causation between education level and obesity. Furthermore, based on the results of these studies, the relevant government departments of public health can proceed to reduce the prevalence of obesity, thereby decreasing mortality and morbidity in the elderly, especially disadvantaged groups with low SES.

## Data Availability

The datasets produced and/or analysed during the present study are available from the corresponding author upon reasonable request.
